# Tissue-Specific Anthocyanin and Polyphenol Content in Sweet Cherry (*Prunus avium* L.): Effects of Freezing and Association with MYB-Based Genetic Variability

**DOI:** 10.3390/molecules31101732

**Published:** 2026-05-19

**Authors:** Csilla Mihályfi, Janka Bedő, Gábor Endre Halász, Hussein G. Daood, Ákos Mendel, Molaligne Medfu Tarekegn, Andrea Kitti Tóth-Lencsés, Zsófia Kovács, András Neményi, Anikó Veres

**Affiliations:** 1 Institute of Genetics and Biotechnology, Szent István Campus, Hungarian University of Agriculture and Life Sciences, Páter Károly utca 1, 2100 Gödöllő, Hungary; mihalyfi.csilla.rita@phd.uni-mate.hu (C.M.); bedo.janka@uni-mate.hu (J.B.); medfu2013@gmail.com (M.M.T.); toth-lencses.andrea.kitti@uni-mate.hu (A.K.T.-L.); kovacs.zsofia@uni-mate.hu (Z.K.); veres.aniko@uni-mate.hu (A.V.); 2 Institute of Environmental Sciences, Department of Environmental Analysis and Technology, Szent István Campus, Hungarian University of Agriculture and Life Sciences, Páter Károly utca 1, 2100 Gödöllő, Hungary; halasz.gabor.endre@uni-mate.hu; 3 Institute of Horticultural Sciences, Szent István Campus, Hungarian University of Agriculture and Life Sciences, Páter Károly utca 1, 2100 Gödöllő, Hungary; daood.hussein@uni-mate.hu; 4 Institute of Landscape Architecture and Garden Art, Ornamental Plant and Green System Management Research Group, Szent István Campus, Hungarian University of Agriculture and Life Sciences, Páter Károly utca 1, 2100 Gödöllő, Hungary; nemenyi.andras.bela@uni-mate.hu

**Keywords:** anthocyanin, polyphenol, *Prunus avium*, CDDP

## Abstract

Sweet cherry (*Prunus avium* L.) is a rich source of phenolic compounds, including anthocyanins and polyphenols, which contribute to fruit quality and nutritional value. However, their distribution across tissues (flesh and skin) and stability under different postharvest freezing treatments remain poorly understood. This study characterized the phenolic profiles of 14 sweet cherry genotypes in different tissues (whole fruit, flesh, and skin) and assessed the effects of freezing storage conditions on compound stability using high-performance liquid chromatography. Results revealed pronounced tissue-specific patterns: most phenolic compounds, particularly total anthocyanins, neochlorogenic acid, rutin, and chlorogenic acid, were more than twofold higher in the skin than in the flesh. Substantial genotype-dependent variability was observed, with certain cultivars exhibiting markedly higher phenolic concentrations. Immediate freezing in liquid nitrogen preserved significantly higher levels of phenolics compared to delayed freezing at −70 °C, where several compounds showed considerable degradation, especially in separated flesh samples. Anthocyanin content increased strongly with pigmentation intensity, with darker-coloured genotypes showing up to a 50-fold higher concentration than lighter-coloured types. Molecular analysis identified MYB-associated polymorphisms corresponding to differences in phenolic accumulation and fruit pigmentation. These findings demonstrate that genotype and tissue type are key determinants of phenolic composition, while immediate postharvest freezing is essential for preserving bioactive compounds. The combined biochemical and molecular approach provides novel insight into the regulation and stability of phenolic compounds in sweet cherry and supports the selection of cultivars with enhanced nutritional quality and improved postharvest performance.

## 1. Introduction

Sweet cherry (*P. avium* L.) belongs to the family *Rosaceae* and represents one of the most economically important fruit species, characterized by high genetic diversity and wide variability among cultivated genotypes [1,2]. The fruit is a rich source of phenolic compounds, including anthocyanins and flavonols, which contribute to its colour, sensory properties, and nutritional value. These bioactive compounds are associated with several health-promoting effects, such as improved metabolic function, reduced inflammation, and enhanced antioxidant capacity [3,4,5,6,7,8,9]. In addition to fresh consumption, sweet cherry by-products, including skins and pomace, have also been identified as valuable sources of phenolics with potential applications in functional foods [4]. 

Among phenolic compounds, anthocyanins, primarily cyanidin derivatives, play a central role in fruit pigmentation and antioxidant activity [8,9,10,11,12]. Hydroxycinnamic acids, such as neochlorogenic acid, chlorogenic acid, and coumaroylquinic acid, represent major non-flavonoid components of the phenolic profile and contribute significantly to antioxidant properties [13,14,15,16]. Flavonols, including rutin and quercetin glycosides, although present in lower concentrations, are also important contributors to the overall phenolic composition and biological activity of sweet cherry [17,18,19]. The concentration of these compounds is highly genotype-dependent and may vary considerably among cultivars [13,15].

A characteristic feature of sweet cherry is the tissue-specific distribution of phenolic compounds, with higher concentrations generally observed in the skin than in the flesh. This pattern is associated with the localization of biosynthetic pathways and the protective role of phenolics against environmental stress factors such as ultraviolet radiation [20,21,22,23,24,25]. Although several studies have described the phenolic composition of whole fruits, fewer investigations have systematically compared the distribution of these compounds between fruit tissues across multiple genotypes, and the extent to which genotype influences tissue-specific accumulation remains insufficiently clarified.

MYB transcription factors play a central role in the regulation of anthocyanin biosynthesis and other secondary metabolic pathways in plants [26]. In sweet cherry, several MYB-associated genes have been linked to pigmentation, fruit development, and phenolic accumulation [27].

Postharvest handling and storage conditions are critical determinants of phenolic stability. Sweet cherries are highly perishable fruits that require low-temperature storage; however, storage conditions may significantly affect the retention or degradation of bioactive compounds. Rapid freezing, especially in liquid nitrogen, can effectively inhibit enzymatic oxidation and preserve metabolite integrity, whereas delayed freezing or suboptimal storage may lead to degradation of phenolic compounds. Although previous studies have examined the effect of storage temperature on phenolic composition [16], comparative analyses focusing on different freezing regimes and their impact on phenolic stability in relation to tissue type and genotype are still limited.

The aim of this study was to characterize the phenolic profiles of 14 sweet cherry genotypes in whole fruit, flesh, and skin tissues using high-performance liquid chromatography, evaluate the effect of immediate and delayed freezing treatments on the stability of phenolic compounds, and investigate the relationship between phenolic composition, fruit pigmentation, and genetic variability using MYB-based CDDP markers. By integrating biochemical and molecular approaches, this study provides new insights into the distribution and stability of phenolic compounds in sweet cherry and contributes to the understanding of factors influencing fruit quality and postharvest preservation.

## 2. Results

We determined the total anthocyanin content and the major phenolic compounds in fourteen cherry varieties using high-pressure liquid chromatography by analyzing whole fruit, flesh, and skin samples. In addition to genotype- and tissue-specific differences, we also evaluated the effect of sample treatment by comparing fruit frozen immediately in liquid nitrogen with fruit frozen at −70 °C. Genetic variability between varieties was examined using MYB-based CDDP primers. The following sections summarize the biochemical patterns obtained and the molecular differences revealed by CDDP analysis.

### 2.1. Comparative High-Pressure Liquid Chromatography Analysis of Sweet Cherry Fruits

**Figure 1:**
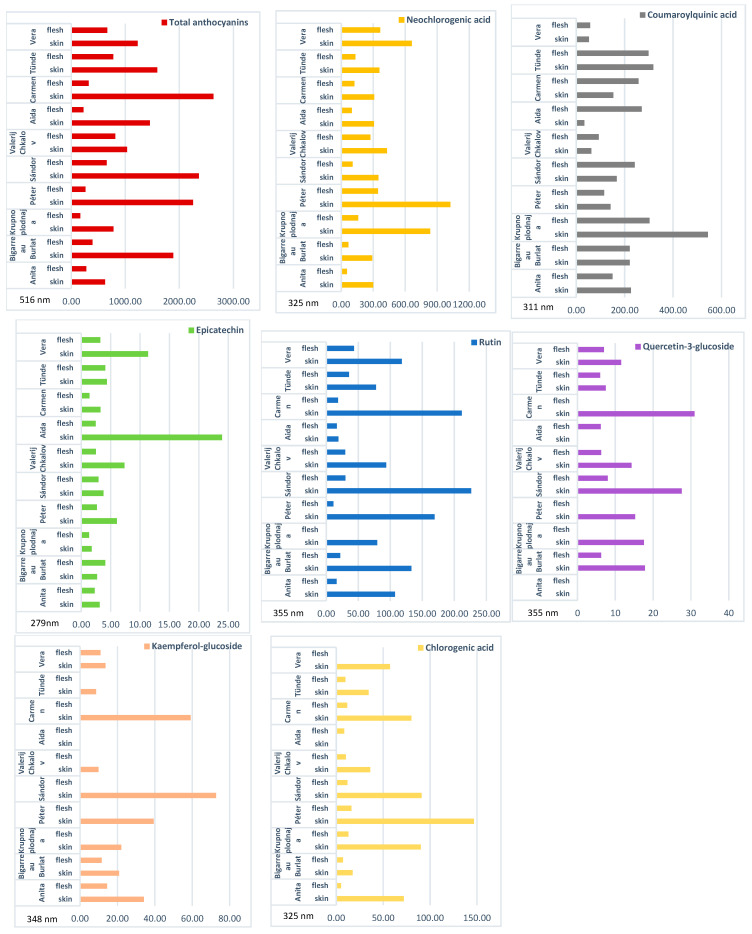
Comparison of the phenolic compound composition in flesh and skin samples of freshly harvested sweet cherry cultivars. Units are given as µg g^−1^ fresh weight (FW).

The concentrations of phenolic compounds across sweet cherry (*P. avium* L.) genotypes are summarized in Figure 1 and Table 1. A consistent tissue-specific pattern was observed, with most phenolic compounds present at higher concentrations in the skin than in the flesh across the majority of genotypes. This trend was particularly pronounced for total anthocyanins, neochlorogenic acid, rutin, and chlorogenic acid, which frequently showed more than twofold higher levels in the skin. In cases where no bars are shown in Figure 1, the respective compounds were not detected in measurable concentrations in the given tissue samples. Substantial genotype-dependent variability was also detected. Cultivars such as ‘Carmen’, ‘Péter’, ‘Sándor’, and ‘Krupnoplodnaja’ exhibited notably elevated phenolic concentrations, especially in the skin, while other genotypes showed more moderate profiles.

Despite the skin-dominant pattern, several compound-specific deviations were identified. Coumaroylquinic acid frequently showed higher concentrations in the flesh in certain genotypes, including ‘Aida’, ‘Carmen’, ‘Sándor’, and ‘Vera’. Similarly, epicatechin and quercetin-3-glucoside were occasionally more abundant in the flesh in specific cultivars, such as ‘Négus’ and ‘Vega’. In addition, some genotypes, including ‘Sárga hibrid’ and ‘Vega’, displayed more variable distribution patterns, with compound localization depending on the specific metabolite. The comparative analysis between flesh and skin samples further confirmed the dominance of phenolic compounds in the outer fruit tissues. In most cultivars, skin samples contained substantially higher concentrations of total anthocyanins and major phenolic acids, supporting the role of the skin as the primary site of phenolic accumulation. Exceptions were observed, particularly for coumaroylquinic acid and certain flavonoids, which exhibited higher concentrations in the flesh in selected genotypes.

### 2.2. Colour Classification

**Figure 2:**
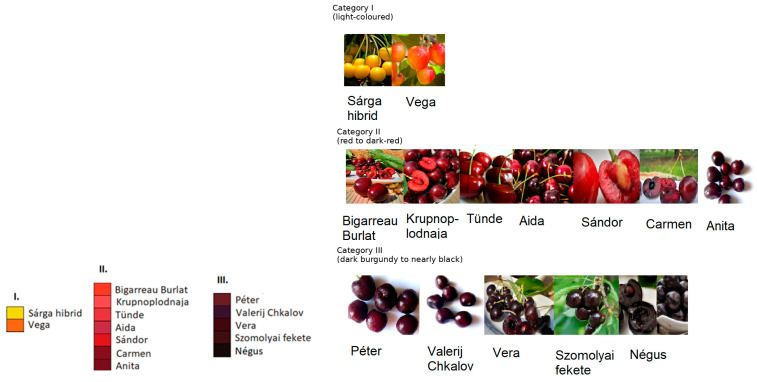
Classification of sweet cherry genotypes according to fruit skin color intensity. Category I: light-coloured; Category II: red to dark-red; Category III: dark burgundy to nearly black. Images of ‘Anita’, ‘Péter’ and ‘Valerij Chkalov’ were taken by the authors, while the remaining cultivar images were obtained from publicly available horticultural websites [28,29,30,31,32,33,34,35,36,37,38].

A standardized colour scale was developed to classify the investigated sweet cherry genotypes based on skin pigmentation intensity (Figure 2). Three categories were defined: Category I included genotypes with light-coloured skin tones; Category II comprised genotypes with red to dark-red flesh and skin; and Category III contained genotypes with dark burgundy to nearly black pigmentation.

### 2.3. Effect of Storage on Fruit Composition

**Figure 3:**
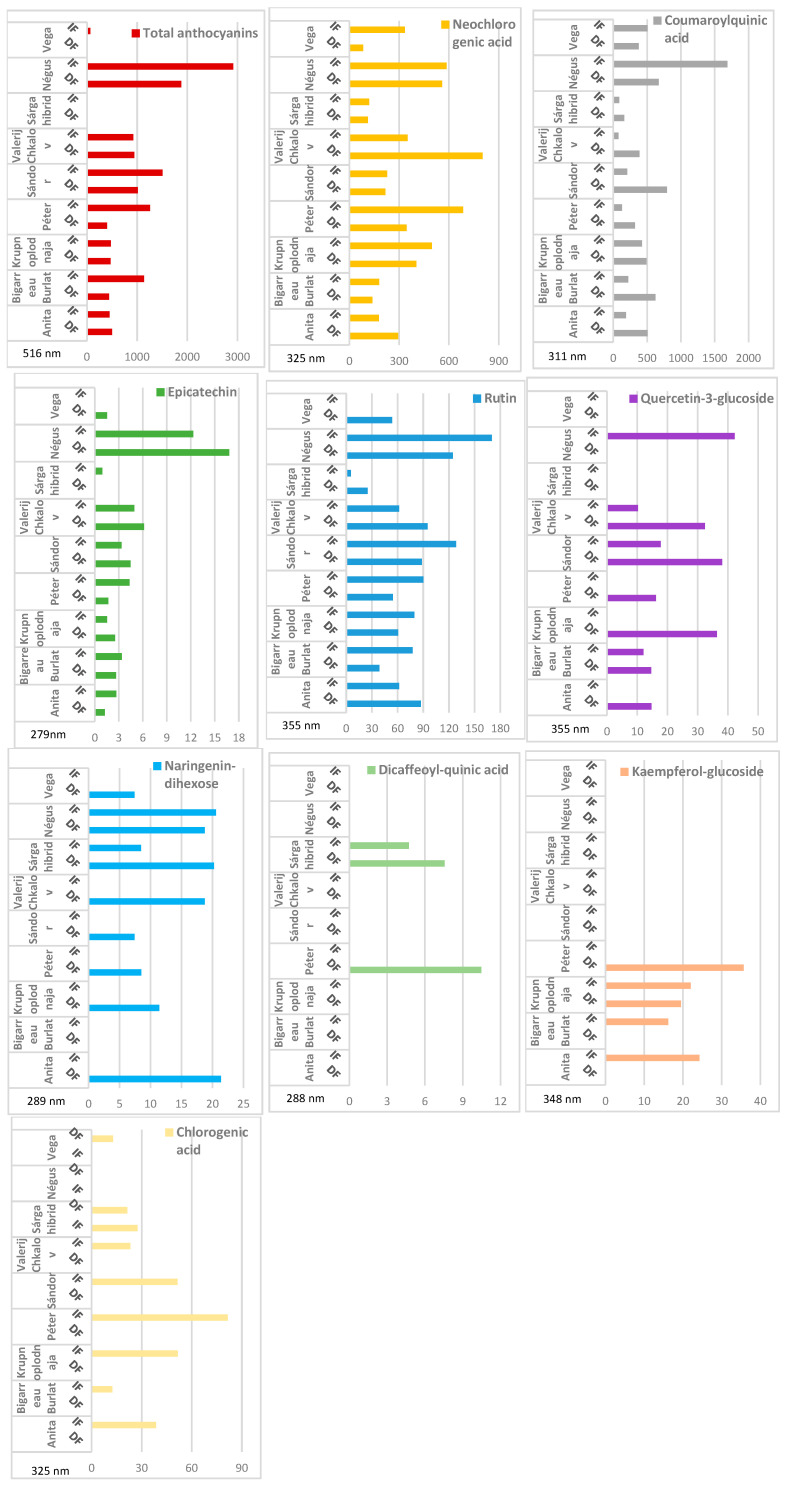
Comparison of the immediately frozen (IF) and delayed frozen (DF) samples. Components of whole fruit samples of ‘Anita’, ‘Bigarreau Burlat’, ‘Krupnoplodnaja’, ‘Péter’, ‘Sándor’ and ‘Valerij Chkalov’ analyzed by high-pressure liquid chromatography. Units are given as µg g^−1^ fresh weight (FW).

The effect of storage conditions on phenolic composition is presented in Table 2 and Figure 3. In cases where no bars are shown in Figure 3, the respective compounds were not detected in measurable concentrations in the given tissue samples. Immediate freezing (IF) resulted in higher concentrations of most phenolic compounds compared to delayed freezing (DF) across the majority of genotypes and compound classes. This trend was particularly evident for total anthocyanins and neochlorogenic acid, which generally showed higher concentrations in IF samples than in DF samples. The differences were especially pronounced in darker-coloured genotypes, indicating more effective preservation of phenolic compounds under immediate freezing conditions. In contrast, DF samples frequently exhibited markedly reduced concentrations, particularly for several flavonoids and anthocyanins. In some DF samples, no bars are shown in Figure 3, indicating that the respective compounds were not detected in measurable concentrations under the applied analytical conditions.

Not all compounds responded uniformly to freezing treatments. Coumaroylquinic acid displayed considerable variability, with no consistent trend between IF and DF treatments. Similarly, minor compounds such as naringenin-dihexose and dicaffeoylquinic acid showed low and inconsistent concentrations, limiting statistical interpretation.

To further evaluate the interaction between freezing treatment and pigmentation intensity, phenolic compound concentrations were compared between Category I and Category III genotypes, which were established based on fruit skin color intensity (Table 3). Under DF conditions, concentrations of most compounds were extremely low or not detectable, particularly in Category I samples. In contrast, IF samples retained substantially higher levels of all measured phenolics.

The difference between treatments was most pronounced for total anthocyanins, which showed a strong increase in Category III under IF conditions. Similar trends were observed for neochlorogenic acid, rutin, and coumaroylquinic acid. These findings confirm that immediate freezing is critical for preserving phenolic integrity, particularly in highly pigmented genotypes.

Although some variability was observed among compounds, the pattern consistently demonstrated superior preservation under IF conditions compared to DF.

**Figure 4:**
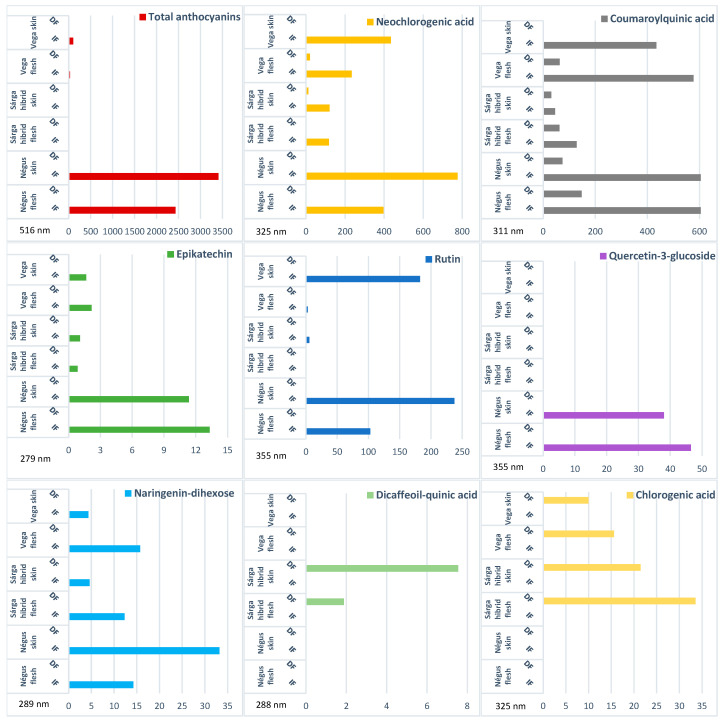
Comparison of the immediately frozen (IF) and delayed frozen (DF) samples. Components of flesh and skin samples of the ’Négus’, ’Sárga hibrid’ and ’Vega’ varieties analyzed by high-pressure liquid chromatography. Units are given as µg g^−1^ fresh weight (FW).

Table 4 and Figure 4 summarize the effects of immediate freezing (IF) and delayed freezing (DF) on phenolic compounds in flesh and skin samples of three representative sweet cherry varieties (‘Négus’, ‘Sárga hibrid’, and ‘Vega’). 

As shown in Table 4, IF preserved detectable levels of most phenolic compounds, whereas DF frequently resulted in non-detectable values, particularly in flesh samples, indicating higher susceptibility to degradation.

The tissue-specific responses are further illustrated in Figure 4. In cases where no bars are shown, the respective compounds were not detected in measurable concentrations in the given tissue samples. Flesh samples showed a marked decline in phenolic content under DF conditions, while skin samples retained relatively higher concentrations, especially under IF treatment.

### 2.4. MYB1 and MYB2 Polymorphisms Associated with Skin Colour Categories and Phenolic Profiles in Sweet Cherry

**Figure 5:**
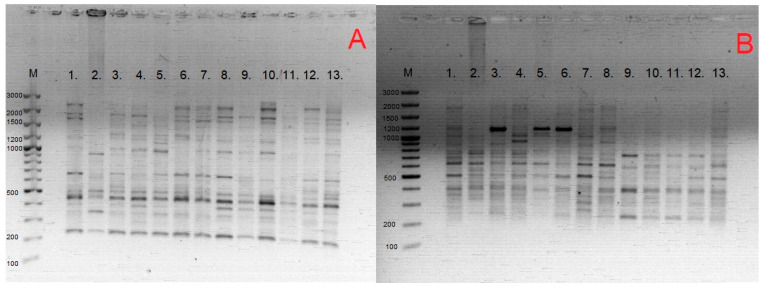
PCR fragments amplified using MYB1 (**A**) and MYB2 (**B**) primers in 13 sweet cherry (*P. avium* L.) genotypes, separated by agarose gel electrophoresis. The MYB1 and MYB2 amplifications were run on separate gels. Lane M: 100 bp DNA ladder (100–3000 bp). Lanes 1–13 correspond to the following cultivars: (1) ‘Vega’, (2) ‘Négus’, (3) ‘Anita’, (4) ‘Bigarreau Burlat’, (5) ‘Carmen’, (6) ‘Krupnoplodnaja’, (7) ‘Aida’, (8) ‘Péter’, (9) ‘Sándor’, (10) ‘Szomolyai fekete’, (11) ‘Valerij Chkalov’, (12) ‘Vera’, and (13) ‘Tünde’.

PCR amplification using MYB1 and MYB2 primers revealed clear polymorphic banding patterns among the examined sweet cherry genotypes (Figure 5, Table 5). The presence or absence of specific fragments indicated genetic variability associated with phenolic accumulation and fruit pigmentation.

Distinct banding patterns were observed among the three colour categories. Genotypes belonging to Category III (dark-coloured fruits) generally exhibited more complex and intense band profiles compared to Categories I and II, suggesting a higher level of genetic variation linked to anthocyanin accumulation.

A consistent relationship was observed between MYB polymorphisms and phenolic composition. Genotypes with higher phenolic concentrations, particularly anthocyanins, tended to display distinct MYB-associated fragments, indicating a possible association between transcriptional regulation and metabolite accumulation.

### 2.5. Hierarchical Cluster Analysis

**Figure 6:**
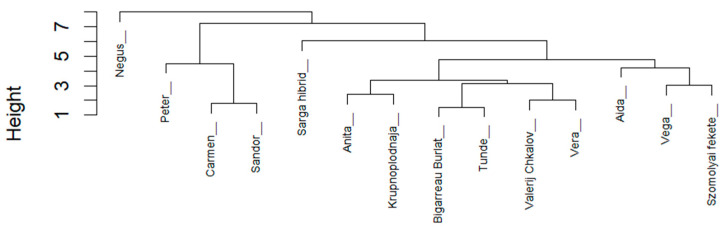
Hierarchical clustering dendrogram (Ward.D2) of sweet cherry genotypes based on phenolic compound concentrations.

Hierarchical cluster analysis (HCA) based on standardized phenolic concentration data revealed distinct grouping patterns among the studied genotypes (Figure 6). The dendrogram showed clear separation of samples according to similarities in their phenolic profiles.

Clusters were generally consistent with the predefined colour categories, with darker-coloured genotypes (Category III) tending to group together, reflecting their higher phenolic and anthocyanin content. In contrast, lighter-coloured genotypes (Category I) formed separate clusters characterized by lower overall phenolic concentrations.

### 2.6. Principal Component Analysis

**Figure 7:**
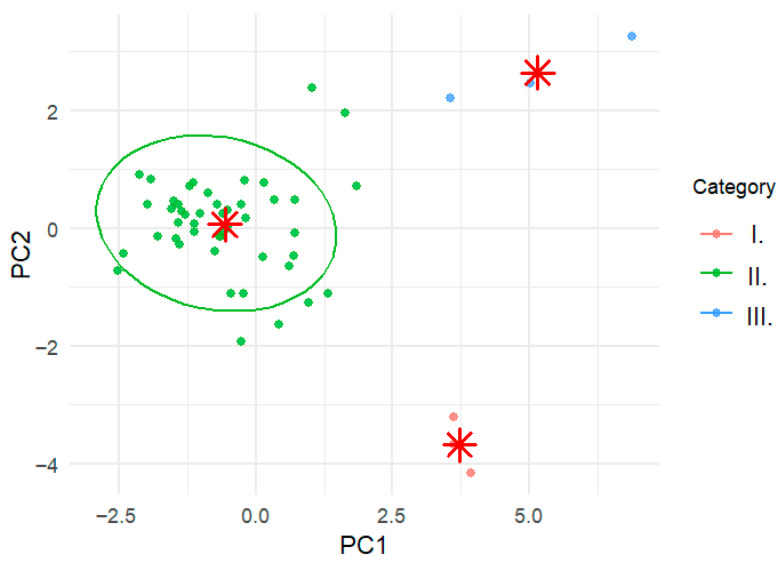
Principal component analysis (PCA) biplot showing k-means clusters based on the phenolic compound profiles of sweet cherry samples. Red stars indicate the cluster centroids identified by the k-means analysis.

Principal component analysis (PCA) was performed to further explore the relationships among genotypes based on their phenolic profiles (Figure 7). The first two principal components accounted for the majority of the total variance, allowing for the visualization of sample distribution in a reduced dimensional space.

The PCA plot revealed a clear separation of genotypes according to phenolic composition, with a noticeable trend corresponding to fruit pigmentation intensity. Genotypes belonging to Category III were generally positioned distinctly from those in Categories I and II, reflecting their higher concentrations of anthocyanins and related phenolic compounds.

Cluster patterns identified by k-means clustering (k = 3) were largely consistent with the PCA distribution, supporting the grouping of genotypes based on phenolic composition. However, some overlap between categories was observed, indicating that phenolic variation is influenced by multiple interacting factors beyond pigmentation alone.

## 3. Discussion

### 3.1. Phenolic Distribution, Genotype Variability and Consistency with Literature

The present study revealed a clear tissue-specific distribution of phenolic compounds in sweet cherry, with most compounds occurring at higher concentrations in the skin than in the flesh. This pattern is consistent with previous studies (Table 5) reporting the accumulation of anthocyanins and flavonoids in epidermal tissues, where they contribute to photoprotection and antioxidant defense. The observed increase in anthocyanin content with increasing pigmentation intensity further supports the strong relationship between fruit colour and phenolic accumulation.

Despite the general skin-dominant trend, notable genotype-dependent variability was observed. Certain compounds, such as coumaroylquinic acid, occasionally showed higher concentrations in the flesh in specific cultivars, indicating that phenolic distribution is influenced by tissue type and by genetic background. Similar genotype-driven differences have been reported in previous studies, where substantial biochemical variability was observed among cultivars even under comparable environmental conditions [49,50].

Differences between whole fruit and separated tissue samples may also partly reflect methodological and seasonal effects. Since samples were collected across multiple harvest years (2019–2024), environmental factors such as temperature, precipitation, and radiation likely contributed to the variability observed. In addition, differences in extraction efficiency between whole fruit and separated tissues may influence the measured phenolic ratios, particularly for compounds predominantly localized in the skin.

Comparison with literature data highlights both consistencies and deviations [51]. While the dominance of anthocyanins, neochlorogenic acid, and rutin in the skin agrees well with previous findings, the magnitude and variability of certain compounds differed from published values. These discrepancies may arise from differences in analytical methods, sample origin, environmental conditions, and genotype-specific metabolic regulation.

The influence of storage conditions on phenolic stability observed in this study is consistent with previous findings [52]. For example, Oliveira et al. [53] reported a decrease in chlorogenic acid and other phenolic compounds during prolonged freezing in peaches, while Zhao et al. [54]. demonstrated a decline in neochlorogenic acid content in ‘Tieton’ sweet cherries during storage. In agreement with these studies, our results showed that delayed freezing led to a significant reduction in anthocyanins and other phenolic compounds, confirming the sensitivity of these metabolites to postharvest conditions.

The stability of flavonoids was strongly dependent on both storage conditions and genotype, with immediate freezing preserving higher concentrations across most compounds. These findings highlight the importance of rapid postharvest stabilization, particularly for maintaining fruit quality and nutritional value. Based on our results, immediate freezing of whole fruit samples is recommended for long-term storage and analytical studies. Our data also provide additional insight into tissue-specific phenolic distribution, which has received limited attention in previous studies that primarily focused on whole fruit analysis. The consistently higher concentrations observed in the skin compared to the flesh support the role of epidermal tissues as the primary site of phenolic accumulation. The observed association between MYB polymorphisms and phenolic composition suggests a potential genetic basis for the variability in metabolite profiles among cultivars. Given the central role of MYB transcription factors in anthocyanin biosynthesis, these results highlight a promising direction for future research aimed at identifying functional markers for breeding programs targeting improved antioxidant capacity and fruit quality [55].

### 3.2. Correlation Between Phenolic Composition and Colour-Dependent Differences Among Sweet Cherry Categories

The relationship between phenolic composition and skin colour intensity is summarized in Table 6. A clear trend was observed across the three colour categories, with significantly lower polyphenol concentrations in Category I genotypes compared to Categories II and III. This difference was particularly pronounced for total anthocyanins, which showed a marked increase with increasing pigmentation intensity, confirming the strong association between fruit colour and anthocyanin accumulation. A similar pattern was observed for several flavonoids, including rutin and quercetin derivatives, which generally increased from lighter to darker genotypes. In contrast, hydroxycinnamic acid derivatives exhibited less pronounced variation among categories, suggesting that these compounds are less directly associated with pigmentation. The intermediate values observed in Category II further support the gradient-like relationship between colour intensity and phenolic composition.

### 3.3. Comparison of Dendrogram and Colour Intensity Categories

The comparison between cluster analysis results and visually defined colour categories revealed a generally consistent relationship between phenolic composition and fruit pigmentation. Genotypes belonging to Category III were clearly grouped together in the dendrogram, reflecting their higher anthocyanin content and overall phenolic richness.

In contrast, Category II genotypes exhibited more heterogeneous clustering, indicating that intermediate pigmentation does not fully capture the underlying biochemical variability. The most pronounced discrepancies were observed in Category I, where certain genotypes (e.g., ‘Vega’) clustered with more highly pigmented samples despite their lighter visual classification.

These findings suggest that while fruit colour is a useful indicator of anthocyanin content, it is not sufficient for comprehensive classification of phenolic composition. Multivariate approaches, such as cluster analysis, provide a more accurate representation of biochemical relationships among genotypes.

### 3.4. Interpretation of Atypical Phenolic Patterns and Proposed Future Analyses

Most phenolic compounds showed higher concentrations in the skin, but several genotype- and compound-specific deviations were observed. These atypical patterns likely reflect differences in metabolic regulation and tissue-specific biosynthesis. For instance, the higher flesh concentrations of coumaroylquinic acid in certain cultivars suggest its role as a phenylpropanoid intermediate that accumulates in the pulp due to differential metabolic flux between tissues.

Similarly, elevated levels of epicatechin and quercetin derivatives in the flesh of selected genotypes may indicate differences in flavonoid transport, storage, or oxidative enzyme activity. In contrast, the consistent accumulation of anthocyanins, rutin, and related compounds in the skin supports their role in photoprotection and stress response, where exposure to environmental factors drives their synthesis.

Storage conditions further influenced phenolic stability. Immediate freezing effectively preserved phenolic compounds, likely due to the rapid inactivation of oxidative enzymes such as polyphenol oxidases and peroxidases. In contrast, delayed freezing resulted in substantial degradation, particularly in flesh tissues, which are more susceptible to oxidation due to the absence of protective epidermal structures.

Genotype-specific responses to storage were also evident, highlighting the complex interaction between metabolic composition and postharvest stability.

## 4. Materials and Methods

### 4.1. Plant Material, Storage Conditions and Characterization of the Investigated Sweet Cherry Cultivars

Fourteen sweet cherry (*P. avium* L.) genotypes were obtained from the Hungarian University of Agriculture and Life Sciences Fruit Growing Research Centre, Érd (Érdi Elvira Major, Hungary), as listed in Table 7. Fruit sampling was carried out over four harvest years (2019, 2021, 2022, and 2024) from the same orchard and the same trees representing each genotype.

### 4.2. Freezing Treatments

To evaluate the effect of storage conditions on phenolic stability, two freezing treatments were applied. In the immediate freezing (IF) treatment, fruit samples were frozen in liquid nitrogen immediately after harvest. In the delayed freezing (DF) treatment, samples were not exposed to liquid nitrogen and were directly stored at −70 °C. Following both treatments, all samples were maintained at −70 °C until further analysis.

### 4.3. High-Pressure Liquid Chromatography Analysis of Phenolic Compounds

#### 4.3.1. Extraction of Phenolics

Frozen sweet cherry tissue (1.5–2.0 g) was homogenized in a mortar with a small amount of quartz sand. Extraction was carried out using 30 mL of solvent consisting of 70% (*v*/*v*) 3% metaphosphoric acid and 30% methanol. Samples were shaken for 15 min at 300 rpm and subsequently stored at 4 °C for 24 h. The extracts were then ultrasonicated for 5 min at 18 °C and centrifuged at 5000× *g* for 5 min. The supernatants were filtered through 0.45 µm hydrophobic PVDF syringe filters into glass vials prior to high-pressure liquid chromatography analysis.

#### 4.3.2. Chromatographic Instrument and Conditions

Phenolic compounds were analyzed using a Hitachi Chromaster Series of high-pressure liquid chromatography system (Hitachi High-Technologies Corporation, Tokyo, Japan) consisting of a Model 5430 diode-array detector (DAD), a Model 5160 gradient pump, a Model 5260 auto sampler, and a Model 5310 column oven. Chromatographic data acquisition and processing were carried out using OpenLab 2.8 software.

Separation of phenolics was performed on a Supelco Ascentis^®^ Express C18-PCP (Merck, Darmstadt, Germany) column (150 × 4.6 mm, 2.7 µm, 90 Å) and a gradient change of acetonitrile in 1% phosphoric acid. The gradient program started with 1% acetonitrile, changed to 30% in 35 min, and turned to 1% in 10 min. Detection of separated phenols was carried out by DAD at wavelengths 190–600 nm according to previously published methods [8,27,56,57,58,59,60,61]. Identification of phenolics was based on using external standard materials (purchased from Sigma-Aldrich via Merck Group, Budapest, Hungary) with comparison of retention and spectral characteristics with those of literature data [8,27,56,57,58,59,60,61]. From each sample, 20 µL were injected into the column. Detection was performed at ten wavelengths corresponding to the analyzed compounds. Total anthocyanins were detected at 516 nm, neochlorogenic acid and chlorogenic acid at 325 nm, coumaroylquinic acid at 311 nm, epicatechin at 279 nm, rutin and quercetin-3-glucoside at 355 nm, naringenin-dihexoside at 289 nm, dicaffeoylquinic acid at 288 nm, and kaempferol-3-glucoside at 348 nm. Total anthocyanin content was calculated as the sum of cyanidin-3-glucoside, cyanidin-3-rutinoside, pelargonidin-3-rutinoside, and peonidin-3-rutinoside. Quantification of compounds was based on a comparison of the peak area with the relevant standard material. The concentration of each compound was expressed as µg g^−1^ fresh weight (FW).

### 4.4. DNA Extraction and CDDP Marker Analysis

#### 4.4.1. DNA Extraction

Leaf samples stored at −70 °C were ground in liquid nitrogen and processed using the DNeasy^®^ Plant Mini Kit (Qiagen, Hilden, Germany), with the addition of polyvinylpyrrolidone (PVP) during grinding. DNA extraction was performed according to the manufacturer’s protocol. DNA concentration and purity were assessed using a NanoDrop ND-1000 spectrophotometer (Thermo Scientific, Wilmington, DE, USA) with NanoDrop 1000 3.8.1 software.

#### 4.4.2. PCR Amplification

CDDP marker analysis was performed according to Collard and Mackill [62] with optimization for Phusion High-Fidelity DNA polymerase. PCR reactions were carried out in a total volume of 10 µL containing 4.2 µL sterile water, 2 µL 5× GC buffer, 0.2 µL 10 mM dNTP mixture, 2 µL DNA template (20 ng µL^−1^), and 1.5 µL primer (100 pg µL^−1^). Amplification was initiated by adding 0.1 µL polymerase. Primer sequences are listed in Table 8.

#### 4.4.3. Gel Electrophoresis

PCR products were separated on 1% agarose gels prepared in 0.5× TBE buffer containing ethidium bromide. Electrophoresis was performed at 110 V for 15 min. A 100 bp DNA ladder was used as a molecular size marker. Gels were visualized under UV light using a MultiImage Light Cabinet (Uvitec Cambridge Ltd., Cambridge, UK).

### 4.5. Statistical Analysis

All statistical analyses were performed using RStudio (version 2024.09.0 + 375, Posit Software, PBC, Boston, MA, USA) running on R (version 4.4.1) and IBM SPSS Statistics (version 29.0, IBM Corp., Armonk, NY, USA). High-pressure liquid chromatography peak area data were converted to concentrations (µg g^−1^ fresh weight, FW) by correcting for extraction solvent volume and sample mass. Data normality and homogeneity of variances were assessed using the Shapiro–Wilk and Levene’s tests, respectively. When assumptions were met, differences were evaluated by one-way ANOVA followed by Tukey’s HSD post hoc test. Otherwise, the Kruskal–Wallis test with Dunn’s post hoc test and Bonferroni correction was applied. For pairwise comparisons, the Wilcoxon rank-sum test was used. Differences among colour categories (I–III) were analyzed using one-way ANOVA with Bonferroni-adjusted pairwise comparisons. Results are expressed as mean ± standard deviation, and statistical significance was set at *p* < 0.05. Data visualization was performed using the ggplot2 package. Multivariate analyses were conducted on standardized (z-score transformed) phenolic concentration data. Hierarchical cluster analysis (HCA) was performed using the Ward.D2 method with Euclidean distance, and k-means clustering (k = 3) was applied to evaluate grouping patterns. Cluster structure was visualized using principal component analysis (PCA).

## 5. Conclusions

This study examined the phenolic composition of fourteen sweet cherry (*P. avium* L.) genotypes by combining biochemical profiling with molecular marker analysis. The results confirmed a strong tissue-specific distribution, with most phenolic compounds occurring at higher concentrations in the skin than in the flesh. Anthocyanins, neochlorogenic acid, rutin, and chlorogenic acid showed particularly pronounced skin accumulation, while several compounds were present only in trace or non-detectable amounts in flesh tissues. Considerable variability was also observed among genotypes, indicating that phenolic composition is influenced not only by tissue type but also by genotype-specific metabolic characteristics.

Storage conditions played a decisive role in phenolic stability. Immediate freezing preserved most compounds more effectively, whereas delayed freezing resulted in substantial degradation, particularly in separated flesh tissues. The differences observed between freezing treatments underline the importance of rapid postharvest stabilization in studies investigating phenolic composition and antioxidant-related compounds. Our results also suggest that whole-fruit storage may provide greater preservation of phenolic integrity during long-term freezing.

The relationship between phenolic composition and fruit colour was reflected in the marked increase in anthocyanin concentration with increasing pigmentation intensity. At the same time, cluster analysis and principal component analysis demonstrated that visual colour categories alone do not fully explain the biochemical variability among sweet cherry genotypes. Certain lighter-coloured cultivars exhibited phenolic profiles similar to darker genotypes, indicating that metabolite composition cannot be predicted exclusively from fruit appearance.

The observed association between MYB polymorphisms and phenolic profiles suggests that genetic factors contribute to differences in metabolite accumulation and pigmentation patterns among cultivars. Although the present study focused primarily on phenolic characterization and marker-associated variability, the results indicate that MYB-related molecular markers may represent useful tools for future breeding strategies aimed at improving antioxidant properties and fruit quality.

Some limitations of the study should also be considered. Samples were collected over multiple harvest years, and environmental factors such as temperature and seasonal conditions may have contributed to part of the observed variability. In addition, the analytical approach focused on selected phenolic compounds and therefore does not fully represent the complete metabolomic composition of sweet cherry fruit.

Future studies should focus on clarifying the genetic regulation of phenolic biosynthesis, sequencing polymorphic MYB-associated fragments, and further investigating genotype-dependent responses to postharvest storage conditions. A more detailed understanding of these processes may contribute to the selection of sweet cherry cultivars with improved nutritional quality, enhanced phenolic stability, and better postharvest performance.

## Figures and Tables

**Table 1:** Mean concentrations of phenolic compounds in sweet cherry flesh and skin samples across colour Categories II and III. Values represent means (µg g^−1^ FW); different superscript letters within rows indicate significant differences at *p* < 0.05 (Bonferroni-adjusted).

Sample Type	Components	Category	
		II.	III.
		Mean	
Flesh	Total anthocyanins	402.78 ^a^	581.42 ^a^
	Neochlorogenic acid	106.502 ^a^	328.217 ^b^
	Coumaroylquinic acid	248.80 ^a^	88.58 ^b^
	Epicatechin	2.649 ^a^	2.790 ^a^
	Rutin	19.818 ^a^	28.073 ^a^
	Quercetin-3-glucoside	3.763 ^a^	4.410 ^a^
	Kaempferol-glucoside	3.71 ^a^	3.65 ^a^
	Chlorogenic acid	9.66 ^a^	8.81 ^a^
Skin	Total anthocyanins	1618.27 ^a^	1505.00 ^a^
	Neochlorogenic acid	392.696 ^a^	705.290 ^a^
	Coumaroylquinic acid	237.42 ^a^	85.34 ^a^
	Epicatechin	6.134 ^a^	8.273 ^a^
	Rutin	122.187 ^a^	127.010 ^a^
	Quercetin-3-glucoside	14.480 ^a^	13.690 ^a^
	Kaempferol-glucoside	31.06 ^a^	20.95 ^a^
	Chlorogenic acid	55.08 ^a^	80.17 ^a^

**Table 2:** Effects of delayed freezing (DF) and immediate freezing (IF) on phenolic compound concentrations in sweet cherry genotypes across colour Categories I, II and III. Values represent means (µg g^−1^ FW); different superscripts within rows denote significant differences at *p* < 0.05 (Bonferroni-adjusted).

			Category		
			I.	II.	III.
			Mean		
Freezing treatment	DF	Total anthocyanins	9.15 ^a^	606.68 ^a^	1077.13 ^a^
		Neochlorogenic acid	97.365 ^a^	263.200 ^ab^	568.963 ^b^
		Coumaroylquinic acid	268.203 ^a^	603.438 ^a^	459.167 ^a^
		Epicatechin	0.763 ^a^	2.728 ^a^	8.217 ^a^
		Rutin	39.238 ^a^	68.660 ^a^	91.303 ^a^
		Quercetin-3-glucoside	0.00 ^a^	25.96 ^a^	16.19 ^a^
		Naringenin-dihexose	13.858 ^a^	10.060 ^a^	15.347 ^a^
		Dicaffeoylquinic acid	3.78 ^a^	0.00 ^a^	3.49 ^a^
		Kaempferol-glucoside	0.00 ^a^	4.88 ^a^	11.88 ^a^
		Chlorogenic acid	17.13 ^a^	0.00 ^b^	0.00 ^b^
	IF	Total anthocyanins	36.34 ^a^	893.45 ^a^	1700.21 ^a^
		Neochlorogenic acid	226.645 ^a^	270.245 ^a^	540.913 ^a^
		Coumaroylquinic acid	296.305 ^a^	259.070 ^a^	631.793 ^a^
		Epicatechin	0.480 ^a^	2.748 ^a^	7.207 ^a^
		Rutin	2.655 ^a^	86.798 ^a^	107.313 ^a^
		Quercetin-3-glucoside	0.00 ^a^	7.45 ^a^	17.51 ^a^
		Naringenin-dihexose	4.230 ^a^	0.000 ^a^	6.860 ^a^
		Dicaffeoylquinic acid	2.36 ^a^	0.00 ^a^	0.00 ^a^
		Kaempferol-glucoside	0.00 ^a^	15.62 ^a^	0.00 ^a^
		Chlorogenic acid	13.76 ^a^	38.52 ^a^	34.94 ^a^

**Table 3:** Effects of delayed freezing (DF) and immediate freezing (IF) on phenolic compound concentrations in sweet cherry genotypes belonging to Categories I and III. Values represent means (µg g^−1^ FW). Different superscript letters within rows indicate significant differences at *p* < 0.05 (Bonferroni-adjusted). ^1^ This category was not included in statistical comparisons because no other valid categories were available for comparison.

	Freezing Treatment			
	DF		IF	
	Category		Category	
	I.	III.	I.	III.
	Mean			
Total anthocyanins	0.00 ^1^	0.00 ^1^	36.34 ^a^	2919.35 ^b^
Neochlorogenic acid	8.53 ^a^	0.00 ^a^	226.64 ^a^	586.47 ^a^
Coumaroylquinic acid	39.23 ^a^	110.40 ^a^	296.30 ^a^	1689.40 ^b^
Epicatechin	0.00 ^1^	0.00 ^1^	1.44 ^a^	12.34 ^b^
Rutin	0.00 ^1^	0.00 ^1^	47.68 ^a^	170.14 ^a^
Quercetin-3-glucoside	0.00 ^1^	0.00 ^1^	0.00 ^a^	42.28 ^b^
Naringenin-dihexose	0.00 ^1^	0.00 ^1^	9.25 ^a^	23.75 ^a^
Dicaffeoylquinic acid	2.36 ^a^	0.00 ^a^	0.00 ^1^	0.00 ^1^
Chlorogenic acid	20.15 ^a^	0.00 ^a^	0.00 ^1^	0.00 ^1^

**Table 4:** Comparative analysis of phenolic compound concentrations in sweet cherry cultivars between the present high-pressure liquid chromatography results and published literature values. Units are given as µg g^−1^ fresh weight (FW).

Component	Literature	Variety	Classification	Literature Data (μg/g)	Our Data (μg/g)
Total anthocyanins	Oancea et al., 2016 [39]	‘Black Gold’	Category III.	50–400	1395.57 ± 946.83
Neochlorogenic acid	Antognoni et al., 2020 [40]	‘Burlat’, ‘Grace Star’, ‘Lapins’	Category II.	150–650	255.83 ± 174.11
Coumaroylquinic acid	Möller B. & Herrmann K. 1983 [41]	‘Maibigarreau’	Category II.	218	311.52 ± 180.54
	Boskov et al., 2022 [42]	‘Carmen’/ ‘Oblacinska’		58.6 ± 3.6	
Epikatechin	De Pascual-Teresa et al., 2000 [43]	N/D	All	54.5	4.68 ± 3.66
	Arts, 2000 [44]			95.3	
Rutin	PrvulovIić et al., 2012 [45]	Sándor, Katalin, Kavics, Peter, Linda, Aida, Carmen, Bigarreau Burlat etc.	Category II	19.4	76.95 ± 59.37
			Category III.		98.56 ± 59.62
Quercetin-3-glucoside	Nunes et al., 2021 [46]	‘Saco’	Category III.	1.6–7.9	21.68 ± 14.27
Naringenin-dihexose	Nunes et al., 2021 [23]	‘Saco’	Category III.	0.1–0.2	19.40 ± 7.57
Dicaffeoylquinic acid	Boskov et al., 2022 [42]	‘Carmen’	Category II.	0.034	0.00
Kaempferol-glucoside	Clodoveo et al., 2023 [47]	‘Lapins’	Category II.	6.6	27.11 ± 19.50
Chlorogenic acid	Nunes et al., 2021 [23]	‘Saco’	Category III.	19	63.94 ± 54.24
	Jakobek et al., 2009 [48]	‘Lapins’	Category II.	18–50	35.72 ± 31.13

**Table 5:** Distribution of PCR fragments amplified with MYB1 and MYB2 primers among sweet cherry colour categories. ‘X’ indicates the presence of the corresponding PCR fragment in the given sweet cherry colour category.

MYB 1 DATA				MYB 2 DATA			
Basepair (bp)	Category I.	Category II.	Category III.	Basepair (bp)	Category I.	Category II.	Category III.
2300		X	X	2500	X	X	
1800		X	X	2000	X	X	X
1700		X		1900		X	X
1400	X		X	1400	X	X	
1300	X		X	900	X	X	X
1200			X	700	X	X	X
1100		X		550		X	X
1000		X	X	450		X	X
900	X	X		380			X
650			X	250	X	X	X
620	X		X				
550		X	X				
400		X	X				
280	X		X				

**Table 6:** Mean (±SE) concentrations of phenolic compounds in the three sweet cherry colour categories classified by skin pigmentation intensity. Values represent means (µg g^−1^ FW); different superscript letters within rows indicate significant differences at *p* < 0.05 (Bonferroni-adjusted).

Categories						
	I.		II.		III.	
Components	Mean	Std. Error of Mean	Mean	Std. Error of Mean	Mean	Std. Error of Mean
Total anthocyanins	16.88 ^a^	8.28	915.81 ^b^	149.94	1116.46 ^b^	227.57
Neochlorogenic acid	127.389 ^a^	36.107	255.826 ^a,b^	37.121	394.536 ^b^	67.419
Coumaroylquinic acid	214.82 ^a^	63.21	311.52 ^a^	38.49	486.59 ^a^	138.33
Epicatechin	0.696 ^a^	0.211	3.790 ^a,b^	0.982	5.631 ^b^	1.155
Rutin	23.434 ^a^	13.861	73.448 ^a^	12.839	73.922 ^a^	15.061
Quercetin-3-glucoside	0.00 ^a^	0.000	11.879 ^b^	2.606	13.011 ^b^	3.440
Naringenin-dihexoside	5.76 ^a^	1.77	1.83 ^a^	1.11	6.79 ^a^	2.33
Dicaffeoylquinic acid	1.55 ^a^	0.76	0.00 ^a^	0.00	0.52 ^a^	0.52
Kaempferol-glucoside	0.00 ^a^	0.00	14.79 ^b^	4.21	5.47 ^a,b^	2.63
Chlorogenic acid	10.17 ^a^	3.20	27.60 ^a^	6.65	25.58 ^a^	10.29

**Table 7:** Sample types of the studied sweet cherry varieties.

Variety	Sample Type
Aida	skin, flesh
Anita	whole, skin, flesh
Bigarreau Burlat	whole, skin, flesh
Carmen	skin, flesh
Krupnoplodnaja	whole, skin, flesh
Négus	whole, skin, flesh
Péter	whole, skin, flesh
Sándor	whole
Sárga hibrid	skin, flesh
Szomolyai fekete	whole, skin, flesh
Tünde	skin, flesh
Valerij Chkalov	whole, skin, flesh
Vega	whole, skin, flesh
Vera	skin, flesh

**Table 8:** Sequences of the CDDP markers based on Collard and Mackill (2009) [62].

Primer	Sequence (5′-3′)	Length (Nucleotides)
MYB 1	GGCAAGGGCTGCCGC	15
MYB 2	GGCAAGGGCTGCCGG	15

## Data Availability

The original contributions presented in this study are included in the article. Further inquiries can be directed to the corresponding author.
